# Generative AI–social media coordinated learning and university students' psychological well-being: dual pathways and the buffering role of perceived support

**DOI:** 10.3389/fpsyg.2026.1788850

**Published:** 2026-03-06

**Authors:** Yu Chen, Guanxi Chen, Jiajun Chen

**Affiliations:** 1 NingboTech University, School of Design, Ningbo, China; 2 Joongbu University, Department of Business Administration, Geumsangun, Republic of Korea; 3 Nantong Institute of Technology, School of Business, Nantong, China

**Keywords:** China, collaborative learning, generative artificial intelligence, psychological distress, psychological well-being

## Abstract

**Introduction:**

Generative artificial intelligence (GAI) and social media are increasingly integrated in university learning, reshaping collaboration and psychological outcomes. This study proposes Intelligent Media–Coordinated Learning Experience (IMCLE) to capture perceived coordination quality in GAI–social media learning, including empowerment effectiveness, collaboration facilitation, feedback visibility, and boundary safety.

**Methods:**

Using two-wave survey data from Chinese university students, we applied an explanation–prediction–necessity strategy integrating PLS-SEM, artificial neural networks (ANN), and necessary condition analysis (NCA). We examined how IMCLE cues relate to collaborative learning (CL), psychological distress (PD), and psychological well-being (PWB), with perceived support (PSup) as a moderator.

**Results:**

All IMCLE cues positively predicted CL, which in turn enhanced PWB. IMCLE cues also positively predicted PD, and PD formed a significant indirect pathway to PWB, indicating that gains and strain may co-occur in highly coordinated learning. PSup weakened the IMCLE.PD relationships and attenuated the PD.PWB association. ANN confirmed the predictive salience of key IMCLE cues and showed nonlinear importance patterns, while NCA identified threshold conditions for high PWB.

**Discussion:**

IMCLE has a dual effect, producing both collaborative gains and psychological strain. The findings inform feedback governance and support infrastructure design to improve students' digital well-being.

## Introduction

In recent years, the deep penetration of generative artificial intelligence (GAI) and social media platforms (i.e., online learning platforms) into higher education has been reshaping how university students acquire knowledge, complete academic tasks, and interact with peers (Gołab-Andrzejak, 2022; Shahzad et al., 2025). In everyday learning, students use GAI for planning and on-demand tutoring, while relying on social media to manage learning routines and check-ins, share notes and resources, seek help and conduct peer review, and coordinate group discussions and task division. As a result, learning is increasingly organized through a collaborative configuration of “individual–tool–platform–group” rather than a linear “individual–textbook” process (Ansari and Khan, 2020; Lang et al., 2025). Because such digitally coordinated learning may shape not only academic efficiency but also students' longer-term psychological experience, psychological well-being (PWB) has become a key outcome indicator for learning quality in the digital era (Bai and Wang, 2025; Liu et al., 2022).

A growing body of research has begun to examine how GAI and social media relate to learning and psychological outcomes. Evidence suggests that GAI can provide immediate support and broaden information access, and that social media can facilitate resource diffusion and interactive communication—potentially increasing engagement and peer connectedness (Alam and Mohanty, 2023). At the same time, highly visible and comparison-prone social environments may amplify overload, evaluative pressure, and emotional depletion, thereby increasing anxiety and psychological distress (PD) (Lin and Lachman, 2024). Notably, emerging studies have started to place GAI and social media within a single framework and use collaborative learning (CL) to explain how technology shapes learning and psychological outcomes (Shahzad et al., 2025), providing useful insights into the “technology–interaction–outcome” chain.

However, three limitations remain and leave a practical and theoretical question insufficiently addressed: why do similar forms of digitally intelligent participation produce divergent psychological consequences? First, many studies still rely on behavioral indicators such as usage frequency, intensity, or intention. These indicators can describe whether students use, or how much they use, but are less informative about how well learning is coordinated—whether coordination feels controllable, feedback is actionable, and boundaries are experienced as safe in day-to-day learning management (Marciano et al., 2024). Second, although process variables such as CL have been introduced, prior work often relies on a single pathway or a single mediator, making it difficult to capture a dual-edged pattern in which resource gains and stress depletion coexist—a possibility that becomes particularly salient in learning contexts characterized by high visibility, uncertain rules, and multitasking demands (Lin and Lachman, 2024). Third, universities increasingly need actionable evidence about what should be improved first and which conditions constitute non-negotiable baselines; yet linear explanations alone rarely provide priority-ranked levers or threshold-based guidance for intervention (Ostic et al., 2021).

To address these gaps, this study proposes Intelligent Media–Coordinated Learning Experience (IMCLE) to capture students' integrated judgments of coordination quality in an environment where GAI and social media are intertwined. This shift moves the focus from whether students use technology to how the coordinated use is experienced. Combining GAI and social media is necessary because, in authentic learning routines, the two rarely operate in isolation: students often socially share, verify, and iteratively refine GAI-generated content, while social interaction and feedback pressure may in turn reshape how students use GAI. Accordingly, learning outcomes are likely driven by the configuration of tool capability × platform mechanisms × interaction structure rather than any single technology *per se*. IMCLE is operationalized into four experiential cues: Empowerment Effectiveness (EE), Collaboration Facilitation (CF), Feedback Visibility (FV), and Boundary Safety (BS). Together, these cues are designed to be measurable and governable, allowing institutions to assess coordination quality beyond usage intensity.

Mechanistically, drawing on a resource gain–stress depletion perspective, we develop a parallel mediation framework linking IMCLE to psychological well-being. On the gain side, a higher-quality coordinated experience should promote collaborative learning (CL), supporting resource sharing and joint problem-solving and thereby enhancing psychological well-being (PWB) (Liu et al., 2022). On the strain side, digitally coordinated learning in high-interaction and high-visibility settings may also be accompanied by heightened demands (e.g., time pressure, evaluative visibility, and multitasking), which can manifest as psychological distress (PD) (Jackson and Serenko, 2023). Importantly, rather than presuming PD to be purely erosive in this context, we treat PD as a strain-related process whose association with PWB is empirically examined within the proposed model. Beyond the mediation mechanism, outcomes may also depend on the support environment. Prior work suggests that supportive contexts shape learners' experiences and adjustment in digital learning settings (Tannert and Gröschner, 2021). We therefore introduce perceived support (PSup) as a boundary condition capturing individuals' confidence that emotional, informational, and instrumental help will be available when needed (Han and Geng, 2023). When perceived support is higher, students may be better able to regulate coordination-related demands; thus, PSup is expected to buffer distress formation (attenuating the IMCLE → PD links) and moderate the PD–PWB link.

This study contributes in three ways. First, it proposes and operationalizes the four-dimensional IMCLE framework, shifting research in intertwined GAI–social media contexts from usage behaviors to coordinated learning experience quality. Second, grounded in a resource gain–stress depletion perspective, it tests a parallel mechanism via collaborative learning and psychological distress and incorporates perceived support to reveal boundary effects of supportive environments (Tannert and Gröschner, 2021; Han and Geng, 2023). Third, by integrating PLS-SEM, ANN, and NCA, the study synthesizes explainability, predictability, and threshold-based evidence, offering more actionable guidance for universities regarding GAI tool integration, social learning platform design, and the governance of students' digital well-being.

## Literature review

### CAC theory and the “resource gain–stress depletion” dual-path framework

The cognition–affect–conation (CAC) theory posits that when individuals are exposed to information and situational cues, they typically form cognitive appraisals first (e.g., judgments of effectiveness, controllability, and predictability), which then elicit corresponding affective/psychological responses and ultimately manifest as relatively stable outcome orientations (Tang et al., 2022). In contexts where digital media and technologies are embedded, this sequence is often used to explain that when cues are perceived as understandable, processable, credible, and controllable, individuals are more likely to develop positive experiences and maintain adaptive psychological states (Huang and Zhang, 2024). Conversely, when cues are accompanied by information overload, evaluative pressure, and normative uncertainty, they are more likely to trigger anxiety and psychological depletion, thereby undermining well-being (Liu et al., 2021). Accordingly, incorporating CAC theory in the present study provides an operational mechanism scaffold to explain the “double-edged” effects of intelligent and digital learning engagement.

Considering the higher-education learning context where generative AI and social media are intertwined, this study follows the overarching “cognition–psychological response–outcome” sequence and further decomposes the intermediate response into two parallel mechanisms to better capture the reality in which empowerment and burden coexist. The first is a resource gain pathway that emphasizes resource acquisition and capability transformation, represented by collaborative learning (CL), which reflects processes of peer support, joint discussion, and co-production in a digitally enabled learning environment (Sharma et al., 2024). This pathway suggests that positive cognitive appraisals are more likely to translate into high-quality interactions and supportive learning, thereby enhancing well-being. The second is a stress depletion pathway that emphasizes burden accumulation and emotional exhaustion, represented by psychological distress (PD), which captures feelings of tension, anxiety, and pressure that may arise under heightened learning visibility, increased information density, and role-related demands (Nieuwoudt, 2021). This pathway posits that negative psychological responses will erode happiness and psychological well-being (PWB).

Within this parallel framework, drawing on prior research on GAI-enabled learning benefits, social collaboration, feedback mechanisms, and psychological safety (Jongsma et al., 2025; Liu et al., 2022; Wang and Fan, 2025; Wang et al., 2025), we propose Intelligent Media–Coordinated Learning Experience (IMCLE) as a set of antecedent cues that captures students' overall cognitive appraisals of a coordinated learning environment shaped jointly by GAI and social media. IMCLE comprises four dimensions—empowerment effectiveness (EE), collaboration facilitation (CF), feedback visibility (FV), and boundary safety (BS)—which together determine whether the environment is perceived as useful, collaboration-friendly, and characterized by feedback that is clear and manageable.

These cues may, on the one hand, activate the resource gain pathway by fostering collaborative learning; on the other hand, they may also trigger the stress depletion pathway through visibility-related pressure and boundary uncertainty, thereby forming a dual-path structure in which the same source produces two opposing effects. In this way, CAC is specified in the present study as a mechanism chain of cues (IMCLE) → parallel internal responses (CL/PD) → outcome orientation (PWB).

### Intelligent media–coordinated learning experience (IMCLE)

As generative AI tools and social media platforms become deeply embedded in learning workflows, university students' learning activities increasingly exhibit an integrated pattern of “tool-assisted learning–coordinated interaction–feedback loops” (Kim et al., 2023). Compared with research traditions that center on technology usage intensity (e.g., frequency of use and usage intention) (Al-Adwan et al., 2023), the most consequential differences in real-world settings often lie in whether students can develop a stable, effective, and controllable learning experience in an environment where GAI and social media are intertwined (Mittal et al., 2024). To more accurately capture this contextual characteristic, this study proposes the concept of Intelligent Media–Coordinated Learning Experience (IMCLE), defined as individuals' overall cognitive appraisal of learning-process quality shaped jointly by “GAI-assisted learning” and “platform-based sharing and interaction.” IMCLE emphasizes not merely whether tools are used, but whether technology and social media are coordinated across the learning task chain—specifically, whether information provision, collaboration organization, feedback generation, and boundary management can be smoothly connected—thereby shaping students' perceived control over the learning process and their sense of psychological safety.

Conceptually, IMCLE can be viewed as a set of antecedent cues that occupies the “cognitive appraisal” position in the CAC sequence. Students draw on cues provided by tools and platforms to judge the usability, effectiveness, and controllability of the learning environment, which in turn triggers subsequent collaborative engagement or stress-related responses. For measurement and mechanism testing, IMCLE is operationalized into four interrelated dimensions. First, empowerment effectiveness reflects whether students perceive that GAI and platform functions deliver substantive learning gains, such as improving comprehension efficiency, reducing task costs, enhancing output quality, or strengthening learning confidence. Second, collaboration facilitation captures whether collaborative relationships are easier to establish and sustain in intelligent media environments—for example, whether resource sharing is convenient, peer support is smooth, and coordination costs for joint task completion are reduced. Third, feedback visibility emphasizes whether the feedback generated through sharing and interaction is timely, clear, and directional—namely, whether students can obtain explicit cues for improvement and a sense of progress from external feedback. Fourth, boundary safety focuses on perceived psychological controllability under conditions of platform visibility and normative uncertainty; it concerns whether students can remain secure and in control when publicly expressing themselves, being evaluated, and leaving digital traces, thereby avoiding persistent tension arising from exposure risks, comparison pressure, or ambiguous rules.

Based on the above conceptualization, IMCLE entails a potential “double-edged” direction of influence. When empowerment effectiveness, collaboration facilitation, and feedback visibility are high and boundary safety is sufficiently ensured, individuals are more likely to enter supportive interactions and collaborative learning processes, generating resource gains. Conversely, when feedback visibility co-occurs with inadequate boundary safety or heightened normative uncertainty, the coordinated environment may be experienced as a stress cue, eliciting psychological distress and producing stress depletion. Therefore, IMCLE not only provides a critical antecedent for explaining why similar digital-intelligent participation can lead to different psychological outcomes, but also establishes the construct foundation for testing the dual-path “resource gain–stress depletion” mechanism.

### Collaborative learning (CL)

Collaborative learning typically refers to a process in which learners, driven by shared goals, co-construct knowledge and enhance competencies through discussion, task division, peer assessment, and resource sharing (Er et al., 2021). Its essence lies not in whether students “learn together,” but in whether interaction generates effective cognitive processing and social support. Compared with individual learning, collaborative learning can reduce comprehension bias and the costs of trial and error through complementary viewpoints, timely feedback, and peer modeling, while strengthening learning motivation and self-efficacy through sustained interaction (Chaudhry et al., 2024). In digitally intelligent learning contexts, the emergence of generative AI and social media has reshaped the conditions under which collaborative learning occurs. On the one hand, GAI can provide rapid information supply, structured articulation, and instant proofreading, offering a “shared draft” that supports discussion and co-production (Lang et al., 2025). On the other hand, platform-based interaction reduces the temporal and spatial costs of collaboration, making help-seeking, peer review, and experience exchange more readily accessible (Wang, 2024). Consequently, collaborative learning is not only a mode of learning but can also be understood as a key psychological and process pathway through which IMCLE translates into positive outcomes.

Within our framework, CL corresponds to the “internal response” component in the CAC sequence and represents a process response oriented toward resource gain. When students form positive cognitive appraisals of an intelligent media–coordinated learning environment (e.g., perceiving it as effective, collaboration-friendly, and characterized by feedback that is clear and controllable), they are more likely to proactively engage in high-quality interactions and develop stable collaborative learning processes. Moreover, collaborative learning is often accompanied by stronger feelings of connectedness, growth, and being supported; these positive experiences can enhance individuals' psychological well-being and subjective happiness. Therefore, incorporating CL into the model helps explain why, within the same learning environment where GAI and social media are intertwined, some students are able to obtain sustained positive psychological returns.

### Psychological distress (PD)

Psychological distress generally refers to a cluster of negative psychological states that arise under stressful circumstances, manifested as tension, anxiety, feelings of pressure, or emotional exhaustion, and characterized by a persistent erosion of daily functioning and subjective well-being (Granieri et al., 2021). In digital learning contexts, psychological distress is not only driven by academic tasks *per se* but may also stem from structural pressures embedded in media environments—for example, increased cognitive load due to higher information density; social comparison and evaluative anxiety induced by heightened content visibility; risk concerns arising from uncertainty in platform rules and academic norms; and boundary conflicts caused by frequent role switching (e.g., studying–socializing–working) (Qi and Yang, 2024). Compared with stressors in traditional classroom settings, such distress tends to be more concealed and more enduring (Ifenthaler et al., 2023).

In the present model, PD also occupies the “internal response” position in the CAC sequence, but it represents the core mechanism of the stress depletion pathway. When certain cues within IMCLE are interpreted as uncontrollable or unsafe (e.g., feedback is visible yet accompanied by evaluative pressure, boundary safety is insufficient, or norms are ambiguous), the digitally coordinated environment may shift from an “empowering resource” to a “source of stress,” thereby eliciting psychological distress. Because psychological distress undermines positive affect, recovery capacity, and life satisfaction, it typically exerts a significant negative effect on happiness/psychological well-being. Incorporating PD into the framework thus captures the other side of digitally intelligent learning: why technologies and platforms, while enhancing efficiency and connectedness, may simultaneously reduce well-being through accumulated stress, leading to substantial differences in outcomes.

### Psychological well-being (PWB)

Psychological well-being (PWB) generally refers to individuals' overall positive evaluation of their life conditions and psychological functioning. It encompasses both affective components such as pleasure and satisfaction, and more enduring functional components such as self-acceptance, positive interpersonal relationships, purpose in life, and a sense of growth. Unlike short-term emotions, PWB emphasizes a relatively sustained psychological state and life quality; therefore, it is commonly used to assess the “long-term psychological outcomes” of changes in digital environments (Baumeister and Robson, 2021). In higher-education settings, students' psychological well-being is shaped not only by academic stressors but also by social connectedness, perceived control, and meaning-making embedded in the learning process (Zhang, 2024). In particular, within digitally intelligent learning environments where generative AI and social media are deeply integrated, the pace of learning activities, feedback structures, and visibility conditions are substantially altered. Such changes may simultaneously generate positive experiences (e.g., capability enhancement and stronger connectedness) and negative experiences (e.g., accumulated stress and psychological depletion), thereby exerting profound influences on PWB.

In the present framework, PWB serves as the ultimate outcome variable, capturing the overall psychological consequences of “empowerment vs. burden” in digitally coordinated learning contexts. Consistent with the CAC sequence, PWB can be viewed as an outcome orientation formed after cognitive appraisals and psychological responses. When IMCLE more readily activates resource gain processes such as collaborative learning, individuals are more likely to experience being supported, feeling capable of growth, and maintaining positive self-evaluations, thus enhancing PWB. Conversely, when experiential cues are more likely to elicit psychological distress and produce stress depletion, PWB may decline. Accordingly, positioning PWB as the focal outcome helps move beyond behavioral indicators such as “willingness to use” or “willingness to share,” and directly addresses a more central issue in digitally intelligent learning governance: how to promote students' digital well-being and psychological health.

### Perceived support (PSup)

Perceived support (PSup) generally refers to individuals' subjective appraisal of the availability of external supportive resources, including emotional support (understanding, comfort, and encouragement), informational support (advice, guidance, and resource provision), and instrumental support (tangible assistance and problem solving). In learning contexts, perceived support may come from peers and teachers, as well as from institutional and platform environments—for example, clear normative guidance, reliable feedback mechanisms, and accessible help-seeking channels (Pan, 2022). Compared with the objective amount of support received, perceived support emphasizes whether individuals believe that help will be available when needed; it is therefore closely associated with coping with stress, emotional stability, and psychological recovery capacity (Aslan et al., 2025). For students operating in digitally intelligent learning environments characterized by high visibility and high uncertainty, support not only represents resource replenishment but also functions as reassurance regarding risks and boundaries, thereby reducing the psychological burden created by uncertainty.

In the present model, incorporating PSup as a boundary condition not only helps identify when and for whom the stress depletion pathway is more salient, but also provides a theoretical basis for universities to reduce psychological risks and enhance students' digital well-being through the design of support systems in digitally intelligent learning settings.

## Hypotheses and model

### Effects of IMCLE on collaborative learning

Prior research suggests that GAI tools and social media-based learning can support collaborative learning by improving information-processing efficiency and facilitating interaction and feedback sharing. However, most studies operationalize these effects using general acceptance-related perceptions (e.g., perceived usefulness and perceived ease of use), and seldom explain how collaborative learning is activated from the perspective of coordination experience quality (Yu et al., 2025). In this study, IMCLE is decomposed into four key cues—empowerment effectiveness, collaboration facilitation, feedback visibility, and boundary safety—which correspond, respectively, to whether students can (a) do tasks better, (b) work together more easily, (c) revise more clearly, and (d) engage more securely. Accordingly, we propose that the four IMCLE dimensions will enhance the level of collaborative learning:

H1a. Empowerment effectiveness (EE) positively influences collaborative learning (CL).

H1b. Collaboration facilitation (CF) positively influences collaborative learning (CL).

H1c. Feedback visibility (FV) positively influences collaborative learning (CL).

H1d. Boundary safety (BS) positively influences collaborative learning (CL).

### Effects of IMCLE on psychological distress

Prior research indicates that in digitally intelligent learning contexts characterized by high visibility and intense interaction, improvements in efficiency, accelerated collaboration, and greater feedback transparency may also entail higher levels of engagement intensity, increased exposure to evaluation, and stronger self-monitoring (Bolourchi and Soleimani, 2021), thereby amplifying psychological load. Accordingly, we posit that the dimensions of IMCLE may increase students' psychological distress:

H2a. Empowerment effectiveness (EE) positively influences psychological distress (PD).

H2b. Collaboration facilitation (CF) positively influences psychological distress (PD).

H2c. Feedback visibility (FV) positively influences psychological distress (PD).

H2d. Boundary safety (BS) positively influences psychological distress (PD).

### Resource gain and stress depletion pathways: collaborative learning, psychological distress, and psychological well-being

Prior research suggests that supportive collaboration facilitates the accumulation of psychological resources—such as positive affect and a sense of growth—thereby enhancing psychological well-being. In contrast, persistent psychological distress consumes attentional and emotion-regulation resources, undermines recovery capacity, and ultimately damages individual well-being (Skalski et al., 2022). Within the “resource gain–stress depletion” framework of the present study, collaborative learning (CL) is treated as a key process manifestation of resource gain, whereas psychological distress (PD) is treated as a key process manifestation of stress depletion. Accordingly, we propose:

H3. Collaborative learning (CL) positively influences psychological well-being (PWB).

H4. Psychological distress (PD) negatively influences psychological well-being (PWB).

Furthermore, this study argues that the effects of digitally intelligent learning experiences on well-being are often realized through process variables rather than operating as simple direct effects. However, existing research has provided relatively limited integrative tests of the two parallel channels—“gain” and “depletion.” Accordingly, we propose that the four IMCLE dimensions (EE, CF, FV, and BS) influence psychological well-being through two concurrent pathways—enhancing collaborative learning and shaping psychological distress—thereby forming a parallel mediation mechanism:

H5a. Collaborative learning (CL) positively mediates the relationship between empowerment effectiveness (EE) and psychological well-being (PWB), such that EE indirectly increases PWB by enhancing CL.

H5b. Collaborative learning (CL) positively mediates the relationship between collaboration facilitation (CF) and psychological well-being (PWB), such that CF indirectly increases PWB by enhancing CL.

H5c. Collaborative learning (CL) positively mediates the relationship between feedback visibility (FV) and psychological well-being (PWB), such that FV indirectly increases PWB by enhancing CL.

H5d. Collaborative learning (CL) positively mediates the relationship between boundary safety (BS) and psychological well-being (PWB), such that BS indirectly increases PWB by enhancing CL.

H6a. Psychological distress (PD) negatively mediates the relationship between empowerment effectiveness (EE) and psychological well-being (PWB), such that EE may indirectly decrease PWB by increasing PD.

H6b. Psychological distress (PD) negatively mediates the relationship between collaboration facilitation (CF) and psychological well-being (PWB), such that CF may indirectly decrease PWB by increasing PD.

H6c. Psychological distress (PD) negatively mediates the relationship between feedback visibility (FV) and psychological well-being (PWB), such that FV may indirectly decrease PWB by increasing PD.

H6d. Psychological distress (PD) negatively mediates the relationship between boundary safety (BS) and psychological well-being (PWB), such that BS may indirectly decrease PWB by increasing PD.

### Boundary conditions of perceived support: buffering and strengthening

Prior research suggests that perceived support reflects individuals' subjective confidence that help is available when needed. Such confidence can strengthen coping resources and buffer stress-related consequences (Zhuang et al., 2025). However, in intelligent media–coordinated learning contexts, whether perceived support conditions the experience-cue → psychological distress linkage still requires empirical verification. We argue that in high-support environments, students can more effectively regulate coordination-related demands and appraise them as manageable, thereby attenuating the extent to which EE/CF/FV/BS are associated with psychological distress, and weakening the impact of distress on psychological well-being. Accordingly, we propose:

H7a. The higher perceived support (PSup), the weaker the positive effect of empowerment effectiveness (EE) on psychological distress (PD; i.e., a buffering effect).

H7b. The higher perceived support (PSup), the weaker the positive effect of collaboration facilitation (CF) on psychological distress (PD).

H7c. The higher perceived support (PSup), the weaker the positive effect of feedback visibility (FV) on psychological distress (PD).

H7d. The higher perceived support (PSup), the weaker the positive effect of boundary safety (BS) on psychological distress (PD).

H8. The higher perceived support (PSup), the weaker the detrimental effect of psychological distress (PD) on psychological well-being (PWB).

**Figure 1:**
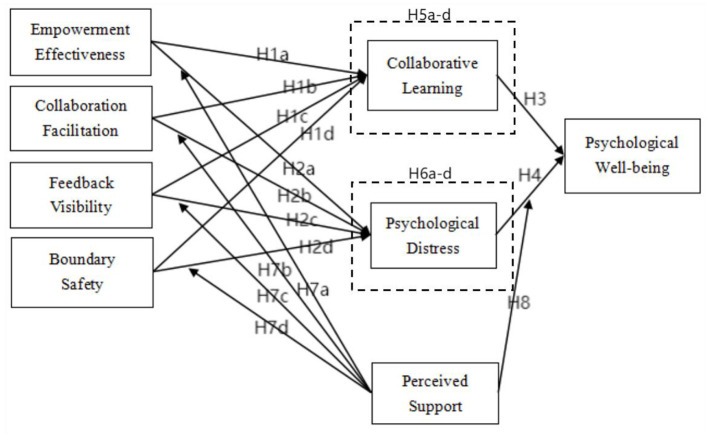
Model.

Based on the above analysis, we propose an integrated conceptual model (Figure 1).

## Methods

### Data collection

This study employed a non-probability sampling strategy, combining convenience sampling with snowball recruitment, to obtain responses from university students in China. Participants were eligible if they (a) were currently enrolled as undergraduate or graduate students, and (b) had used generative AI tools and social media/online learning platforms for routine learning activities during the current semester (e.g., planning, tutoring, information sharing, peer discussion, and group coordination). Participants who did not meet these criteria, provided incomplete responses, or failed attention/quality checks were excluded.

Recruitment was initiated by the research team with assistance from student coordinators, using a combination of online and offline approaches. Online recruitment was conducted through study-related groups on major social networking platforms (e.g., WeChat and Xiaohongshu). The research team first invited students from one public university, two private universities, and several additional institutions to access the survey link, and then encouraged them to forward the link to classmates and acquaintances (snowball expansion). Although this approach enables efficient access to a digitally active student population, it may limit the representativeness of the sample. Future studies may consider adopting more rigorous probability-based strategies (e.g., random sampling or stratified sampling) to enhance representativeness.

In total, 613 matched paired cases were returned across two waves. After data screening, 500 valid matched cases were retained for analysis. Respondents were mainly drawn from universities in Zhejiang Province and Jiangsu Province.

To reduce potential common method bias, data were collected at two time points following time-lagged measurement recommendations. At Time 1 (T1), data collection took place in October 2025 (07/10/2025–14/10/2025). Participants completed a questionnaire capturing demographic information (gender, age, academic year, and major) and the four dimensions of Intelligent Media–Coordinated Learning Experience (IMCLE; empowerment effectiveness, collaboration facilitation, feedback visibility, and boundary safety). Approximately 1 month later, at Time 2 (T2), data collection took place in November 2025 (07/11/2025–14/11/2025). The second questionnaire was distributed only to respondents who had completed T1 and assessed psychological distress (PD), perceived support (PSup), and psychological well-being (PWB).

To enable accurate matching across the two waves while preserving anonymity, participants generated a self-created anonymous matching code at T1 (e.g., “the last four digits of the phone number + birth month and day”) and re-entered the same code at T2. The code was used solely for pairing responses and was anonymized and securely stored. Before participation, respondents reviewed an informed-consent statement indicating voluntary participation, the right to withdraw at any time, academic-only data use, and anonymous reporting.

Data screening and quality control. Matched responses were screened using multiple criteria. First, cases with missing key variables or unmatched codes were removed. Second, we excluded responses showing straight-lining or highly patterned answering across scales. Third, we checked for implausibly short completion times (e.g., below a minimum threshold) and inconsistent responses to attention-check items (if applicable). After these procedures, 500 valid matched cases were retained.

All constructs were measured using established multi-item scales adapted to the learning context (items available in Supplementary material). Responses were recorded on a Likert-type scale (e.g., 1 = strongly disagree to 5/7 = strongly agree). IMCLE was operationalized into four dimensions: empowerment effectiveness (EE), collaboration facilitation (CF), feedback visibility (FV), and boundary safety (BS). Psychological distress (PD), perceived support (PSup), and psychological well-being (PWB) were assessed at (T2).

We tested the hypotheses using PLS-SEM. The model was estimated in SmartPLS 4.1.1.4. In addition, ANN was used to assess predictive importance, and NCA was applied to identify necessity-based thresholds, forming an explanation–prediction–necessity evidence chain.

Although IMCLE comprises four dimensions, we conceptualize it as an experience-quality latent variable that is reflected in empowerment effectiveness, collaboration facilitation, feedback visibility, and boundary safety, rather than being mechanically “formed” by them. This aligns with guidance on reflective–reflective higher-order constructs in PLS-SEM, which are appropriate when lower-order dimensions represent coherent manifestations of an overarching concept and are expected to covary as that concept changes. In our framework, improvements in coordinated learning experience quality should be expressed through convergent shifts across these four experiential cues, consistent with reflective measurement logic (Ringle et al., 2024).

By contrast, a formative specification is more suitable when indicators are relatively independent components whose contributions can vary without co-moving and where omitting a component would alter the construct domain. Finally, given ongoing methodological discussion about reflective measurement in PLS-SEM, our specification is explicitly theory-driven and evaluated using recommended reflective assessment criteria (Guenther et al., 2025).

### Scale design

We developed the measurement scales in this study by taking into account the specific research context. Specifically, to measure Intelligent Media–Coordinated Learning Experience (IMCLE), we developed a 20-item scale drawing on prior studies (Basri, 2024; Kashive et al., 2020; Mavri et al., 2020; Oswal et al., 2021; Ter Hoeven et al., 2021; Wisniewski et al., 2020; Zhou and Chen, 2021). The scale comprises four dimensions: authenticity, presence, narrative guidability, and system usability. Collaborative learning (CL) was adapted from Yilmaz and Karaoglan Yilmaz (2020) and Chatterjee and Correia (2020), including five items. Psychological distress (PD) was adapted from Feng et al. (2020) and Every-Palmer et al. (2020), including five items. Psychological well-being (PWB) was adapted from Morales-Rodríguez et al. (2020), including five items. Perceived support (PSup) was adapted from Sun et al. (2022) and Brunsting et al. (2021), including five items (Table 1).

**Table 1:** Measurement scale.

**Initial dimension**	**Code**	**Item description**
Empowerment effectiveness (EE)	EE1	The combination of GAI and social media significantly improves my efficiency in completing learning tasks
	EE2	This collaborative approach helps me better understand learning content or grasp key points
	EE3	It can improve the quality of my assignments/reports/projects
	EE4	This collaborative approach gives me more confidence in solving learning problems
	EE5	I can better control my learning pace and process through this collaborative approach
Collaboration facilitation (CF)	CA1	In this collaborative environment, I can easily find suitable peers for learning collaboration
	CA2	Initiating or joining collaboration (teamwork, task division, group discussion) is very convenient for me
	CA3	Communication and sharing needed for collaboration (files/links/materials) are easy to access
	CA4	We can easily coordinate time, tasks, and progress to advance the collaboration
	CA5	Even without meeting offline, we can effectively engage in collaborative learning
Feedback visibility (FV)	FV1	In this collaborative environment, I can receive timely feedback related to learning tasks
	FV2	The feedback I receive is usually specific and clear, pointing out areas that need improvement
	FV3	Feedback is presented clearly (key points, logic, and modifications are easy to understand)
	FV4	I can track the source and basis of feedback (which content/discussion corresponds to the suggestions)
	FV5	I can turn the feedback I receive into actionable revisions and optimizations
Boundary safety (BS)	BS1	I know which content is appropriate to share publicly and which is not
	BS2	I can control my privacy and the scope of information exposure (account, content, visibility)
	BS3	I do not overly worry about being criticized or attacked when asking questions, presenting, or sharing
	BS4	The platform/course provides clear rules and boundaries for using GAI and sharing outcomes
	BS5	When disputes or misunderstandings arise, I know how to protect myself and resolve the issues
Collaborative learning (CL)	CL1	I engage in discussions with peers to reach a consensus in learning
	CL2	We divide tasks and collaborate to complete the same learning output (report/project/plan)
	CL3	I provide constructive feedback to my peers, and they also give me effective feedback
	CL4	I adopt the suggestions of peers/groups to improve my learning outcomes
	CL5	Collaborating with peers helps me gain a deeper understanding of knowledge and solve difficult problems
Psychological distress (PD)	PD1	In the past month, this collaborative learning has caused me noticeable stress or tension
	PD2	I often feel anxious about falling behind the pace of information/tasks
	PD3	I often feel overwhelmed by too much information, causing a heavy mental burden
	PD4	I feel uneasy due to comparisons, evaluations, or uncertainty
	PD5	After learning, I often feel exhausted or emotionally drained
Psychological well-being (PWB)	PWB1	In the past month, my overall mental state has been good, and my mood has been positive
	PWB2	I maintain a strong sense of control and stability in both my learning and life
	PWB3	I feel that I am continually growing in my learning and remain hopeful for the future
	PWB4	When facing difficulties, I can quickly adjust and return to a normal state
	PWB5	Overall, I am satisfied with my current quality of life
Perceived support (PSup)	PS1	When encountering difficulties in learning, there is always someone willing to listen and offer encouragement
	PS2	I can receive useful advice or learning guidance from peers/teachers
	PS3	I can obtain practical help to solve specific problems in learning tasks (resources/tools/methods)
	PS4	The school/course provides clear guidelines and support for using GAI and online collaboration
	PS5	When I feel pressure or distress, I know where to seek help and receive a response

### SEM–ANN–NCA method

**Stage 1:** PLS-SEM (Testing Linear Sufficiency)

First, partial least squares structural equation modeling (PLS-SEM) was employed to estimate the proposed “generative AI–social media interaction–psychological well-being” framework. The analysis incorporated exogenous variables (generative AI and social media), mediators (collaborative learning and psychological distress), and psychological well-being as the outcome variable. We assessed measurement reliability and validity, discriminant validity, common method bias, path significance, and model fit to reveal the linear effect paths and their statistical significance.

**Stage 2:** ANN (Nonlinear Prediction and Importance Ranking)

Building on the PLS-SEM results, latent variable scores were entered into a multilayer perceptron (MLP) artificial neural network to perform predictive modeling. Ten-fold cross-validation and the root mean square error (RMSE) criterion were applied to reduce the risk of overfitting. Sensitivity analysis was then used to calculate the relative contribution of each antecedent, supplementing and benchmarking the linear importance ranking obtained from PLS-SEM, and identifying potential nonlinear structures and key predictors.

**Stage 3:** NCA (Identifying Necessity Constraints)

Finally, necessary condition analysis (NCA) was conducted to examine whether any key conditions exhibit “threshold effects” for psychological well-being. The CE-FDH and CR-FDH techniques were used to estimate necessity effect sizes and bottleneck levels, identifying whether any single variable constitutes an indispensable requirement for achieving high levels of psychological well-being.

By integrating the linear mechanism evidence from SEM, the nonlinear predictive evidence from ANN, and the necessity validation from NCA, we established a triangulated analytic system that strengthens the comprehensiveness and robustness of the conclusions in terms of explanatory power, predictive power, and the identification of contextual constraints.

## Results

### Data analysis and hypothesis testing

Hypotheses were tested using partial least squares structural equation modeling (PLS-SEM) in SmartPLS 4.1.1.4 (Cheah et al., 2020).

### Measurement model assessment

#### Reliability and convergent validity

The measurement scales in this study demonstrated satisfactory reliability and convergent validity. All standardized factor loadings for the latent constructs exceeded 0.70, indicating adequate indicator reliability. Cronbach's alpha values ranged largely from 0.79 to 0.86, suggesting good internal consistency. Composite reliability (CR) values were all above 0.80, further supporting scale reliability. In addition, the average variance extracted (AVE) for all constructs exceeded 0.70, meeting the recommended threshold for convergent validity. Overall, these results indicate that the measurement instruments used in this study exhibit strong reliability and convergent validity (Table 2).

**Table 2:** Cronbach's α, AVE and CR values.

**Latent variable**	**Observed variable**	**Factor loading**	**Cronbach's α**	**CR**	**AVE**
Empowerment effectiveness (EE)	EE1	0.814	0.839	0.88601	0.608775503
	EE2	0.772			
	EE3	0.799			
	EE4	0.743			
	EE5	0.771			
Collaboration facilitation (CF)	CF1	0.807	0.818	0.873462	0.581545417
	CF2	0.840			
	CF3	0.764			
	CF4	0.725			
	CF5	0.663			
Feedback visibility (FV)	FV1	0.714	0.869	0.904877	0.656
	FV2	0.829			
	FV3	0.837			
	FV4	0.821			
	FV5	0.843			
Boundary safety (BS)	BS1	0.733	0.857	0.897251	0.63652021
	BS2	0.770			
	BS3	0.854			
	BS4	0.818			
	BS5	0.808			
Collaborative learning (CL)	CL1	0.647	0.797	0.860661	0.555
	CL2	0.757			
	CL3	0.707			
	CL4	0.844			
	CL5	0.755			
Psychological distress (PD)	PD1	0.805	0.769	0.844319	0.524
	PD2	0.582			
	PD3	0.728			
	PD4	0.817			
	PD5	0.662			
Psychological well-being (PWB)	PWB1	0.760	0.840	0.886268	0.609
	PWB2	0.777			
	PWB3	0.783			
	PWB4	0.786			
	PWB5	0.796			
Perceived support (PSup)	PS1	0.778	0.827	0.878599	0.592
	PS2	0.717			
	PS3	0.818			
	PS4	0.740			
	PS5	0.790			

#### Discriminant validity

The results indicate satisfactory discriminant validity. For each latent construct, the square root of AVE was greater than its correlations with other constructs, suggesting adequate distinctiveness among the constructs and effective differentiation across conceptual domains. This finding confirms that the measured latent variables can be clearly distinguished, thereby avoiding conceptual overlap (Table 3).

**Table 3:** Discriminant validity.

**Construct**	**BS**	**CF**	**CL**	**EE**	**FV**	**PD**	**PS**
CA	0.25						
CL	0.544	0.687					
EE	0.325	0.349	0.624				
FV	0.399	0.432	0.651	0.36			
PD	0.468	0.438	0.489	0.401	0.465		
PS	0.428	0.232	0.375	0.175	0.245	0.181	
PWB	0.587	0.495	0.75	0.488	0.573	0.717	0.529

### Common method bias

To assess potential common method bias, this study employed variance inflation factors (VIFs). The results showed that all VIF values ranged from 1.01 to 1.42, which is well below the recommended cutoff of 5 (Hair et al., 2019), indicating that common method bias is unlikely to be a serious concern in this study.

### Structural model evaluation

**Figure 2:**
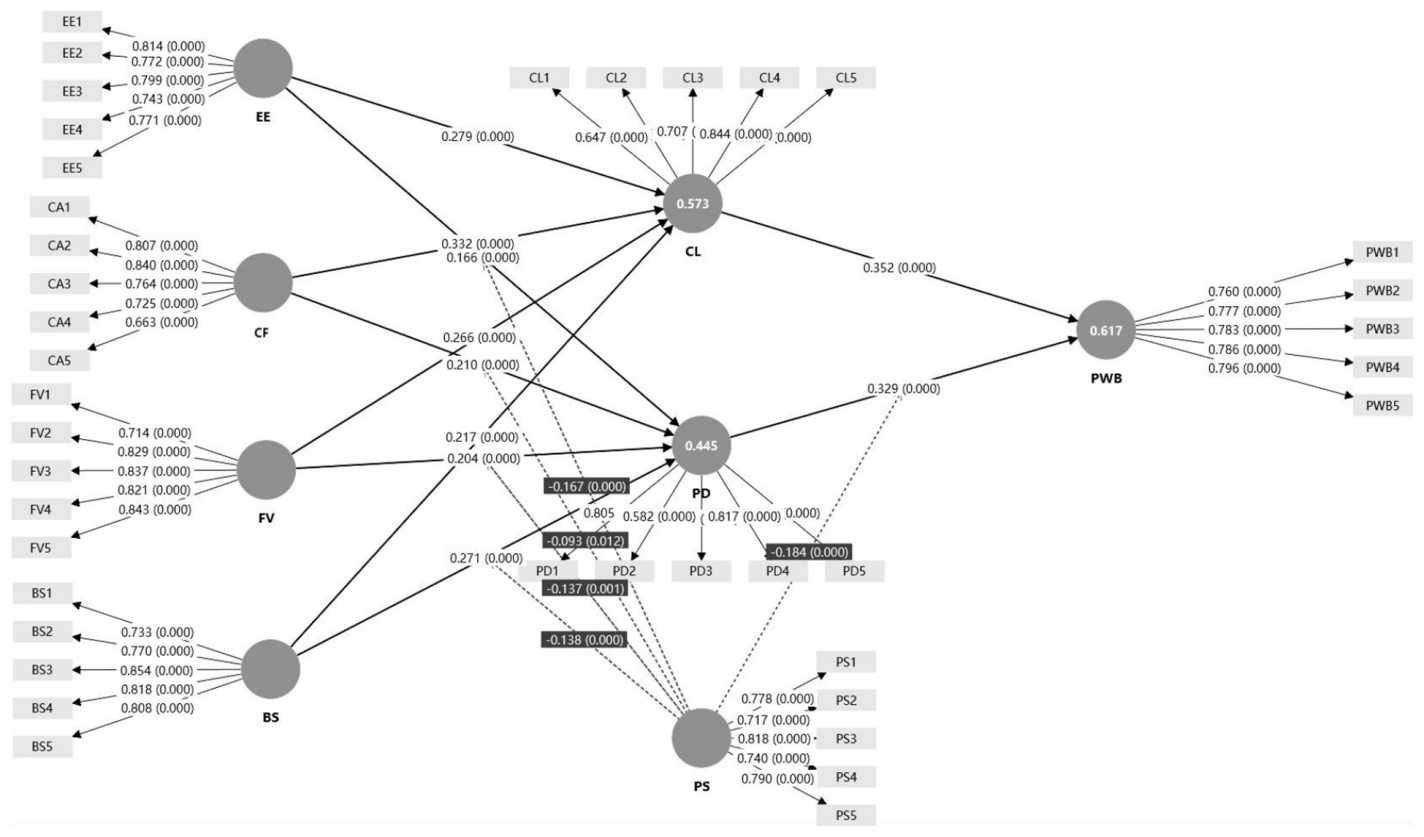
Structural equation modeling analysis results.

This study employed SmartPLS 4.1.1.4 to conduct structural equation modeling; the specific model specification and results are presented in Figure 2.

#### Model fit evaluation

Multiple indicators were used to evaluate model fit. The *R*^2^ values for the endogenous constructs—collaborative learning (CL), psychological distress (PD), and psychological well-being (PWB)—were 0.573, 0.445, and 0.617, respectively, indicating that the model explains a substantial proportion of variance. The adjusted *R*^2^ values were 0.569, 0.435, and 0.614, further supporting the robustness of the model. In addition, the SRMR values for the saturated model and the estimated model were 0.042 and 0.044, both below the recommended threshold of 0.08, suggesting good model fit (Tables 4, 5).

**Table 4:** *R*^2^.

**Endogenous construct**	***R*-square**	***R*-square adjusted**
CL	0.573	0.569
PD	0.445	0.435
PWB	0.617	0.614

**Table 5:** SRMR values.

**Fit index**	**Saturated model**	**Estimated model**
SRMR	0.042	0.044

#### Path analysis

This study used SmartPLS to estimate the path effects and test the proposed hypotheses. The results are presented in Table 6.

**Table 6:** Path analysis.

**Hypothesis**	**Path**	**β**	***Se***	***t***	***p***
H1a	EE → CL	0.279	0.030	9.152	0.000
H1b	CF → CL	0.332	0.034	9.641	0.000
H1c	FV → CL	0.266	0.033	8.039	0.000
H1d	BS → CL	0.217	0.030	7.142	0.000
H2a	EE → PD	0.166	0.037	4.492	0.000
H2b	CF → PD	0.210	0.040	5.196	0.000
H2c	FV → PD	0.204	0.039	5.179	0.000
H2d	BS → PD	0.271	0.041	6.544	0.000
H3	CL → PWB	0.352	0.031	11.478	0.000
H4	PD → PWB	0.329	0.031	10.443	0.000

The path analysis results provide support for most of the proposed direct-effect hypotheses. First, the four IMCLE dimensions all showed significant positive effects on collaborative learning (CL). Specifically, empowerment effectiveness (EE) positively predicted CL (β = 0.279, *SE* = 0.030, *t* = 9.152, *p* < 0.001), supporting H1a. Collaboration facilitation (CF) also had a significant positive effect on CL (β = 0.332, *SE* = 0.034, *t* = 9.641, *p* < 0.001), supporting H1b. Feedback visibility (FV) was positively associated with CL (β = 0.266, *SE* = 0.033, *t* = 8.039, *p* < 0.001), supporting H1c. Boundary safety (BS) likewise exerted a significant positive effect on CL (β = 0.217, *SE* = 0.030, *t* = 7.142, *p* < 0.001), supporting H1d.

Regarding psychological distress (PD), all four IMCLE dimensions exhibited significant positive effects. EE significantly increased PD (β = 0.166, *SE* = 0.037, *t* = 4.492, *p* < 0.001), supporting H2a. Similarly, CF showed a significant positive association with PD (β = 0.210, *SE* = 0.040, *t* = 5.196, *p* < 0.001), supporting H2b. FV also positively predicted PD (β = 0.204, *SE* = 0.039, *t* = 5.179, *p* < 0.001), supporting H2c. BS had the strongest positive effect on PD among the four cues (β = 0.271, *SE* = 0.041, *t* = 6.544, *p* < 0.001), supporting H2d.

Finally, CL was positively related to psychological well-being (PWB; β = 0.352, *SE* = 0.031, *t* = 11.478, *p* < 0.001), supporting H3. In contrast, the direct path from PD to PWB was positive and significant (β = 0.329, *SE* = 0.031, *t* = 10.443, *p* < 0.001). This direction is inconsistent with the hypothesized negative relationship in H4; therefore, H4 is not supported by the path analysis results reported in Table 6.

#### Mediation effects and moderating effect test

This study used SmartPLS to test the mediation effects. The results are reported in Table 7.

**Table 7:** Mediation effects and moderating effect analysis.

**Hypothesis**	**Path**	**β**	***Se***	***t***	***p***
H5a	EE → CL → PWB	0.098	0.014	7.187	0.000
H5b	CF → CL → PWB	0.117	0.016	7.488	0.000
H5c	FV → CL → PWB	0.094	0.014	6.536	0.000
H5d	BS → CL → PWB	0.076	0.013	5.726	0.000
H6a	EE → PD → PWB	0.055	0.014	4.001	0.000
H6b	CF → PD → PWB	0.069	0.015	4.490	0.000
H6c	FV → PD → PWB	0.067	0.015	4.381	0.000
H6d	BS → PD → PWB	0.089	0.016	5.416	0.000
H7a	PS × EE → PD	−0.167	0.039	4.283	0.000
H7b	PS × CF → PD	−0.093	0.037	2.525	0.012
H7c	PS × FV → PD	−0.137	0.040	3.458	0.001
H7d	PS × BS → PD	−0.138	0.039	3.514	0.000
H8	PS × PD → PWB	−0.184	0.031	5.989	0.000
H7a + H6a	PS × EE → PD → PWB	−0.055	0.013	4.114	0.000
H7b + H6b	PS × CF → PD → PWB	−0.031	0.012	2.462	0.014
H7c + H6c	PS × FV → PD → PWB	−0.045	0.014	3.324	0.001
H7d + H6d	PS × BS → PD → PWB	−0.045	0.014	3.308	0.001

The mediation analysis indicates that collaborative learning (CL) serves as a significant positive mediator between the IMCLE dimensions and psychological well-being (PWB). Specifically, the indirect effect of empowerment effectiveness (EE) on PWB via CL was positive and significant (EE → CL → PWB: β = 0.098, *SE* = 0.014, *t* = 7.187, *p* < 0.001), supporting H5a. Similarly, collaboration facilitation (CF) showed a significant positive indirect effect through CL (CF → CL → PWB: β = 0.117, *SE* = 0.016, *t* = 7.488, *p* < 0.001), supporting H5b. The CL-mediated effects were also significant for feedback visibility (FV; FV → CL → PWB: β = 0.094, *SE* = 0.014, *t* = 6.536, *p* < 0.001), supporting H5c, and for boundary safety (BS; BS → CL → PWB: β = 0.076, *SE* = 0.013, *t* = 5.726, *p* < 0.001), supporting H5d. Overall, these findings suggest that higher-quality coordinated learning experiences translate into improved well-being partly by strengthening collaborative learning processes.

Regarding the psychological distress (PD) mediation channel, all four IMCLE dimensions also exhibited statistically significant indirect effects on PWB through PD: EE → PD → PWB (β = 0.055, *SE* = 0.014, *t* = 4.001, *p* < 0.001), CF → PD → PWB (β = 0.069, *SE* = 0.015, *t* = 4.490, *p* < 0.001), FV → PD → PWB (β = 0.067, *SE* = 0.015, *t* = 4.381, *p* < 0.001), and BS → PD → PWB (β = 0.089, *SE* = 0.016, *t* = 5.416, *p* < 0.001). These results confirm that PD constitutes a significant mediating pathway. However, the direction of these PD-mediated effects is positive in Table 7, which is inconsistent with the “negative mediation” wording in H6a–H6d (i.e., “indirectly decrease PWB”). Statistically, the mediation via PD is supported in significance terms, but the hypothesized direction is not supported as stated.

The moderation analysis demonstrates that perceived support (PSup) significantly buffers the relationships between IMCLE dimensions and psychological distress. Specifically, PSup significantly moderated the path from EE to PD (PS × EE → PD: β = −0.167, *SE* = 0.039, *t* = 4.283, *p* < 0.001), supporting H7a. Likewise, PSup weakened the positive association between CF and PD (PS × CF → PD: β = −0.093, *SE* = 0.037, *t* = 2.525, *p* = 0.012), supporting H7b. Similar buffering effects were observed for FV (PS × FV → PD: β = −0.137, *SE* = 0.040, *t* = 3.458, *p* = 0.001), supporting H7c, and for BS (PS × BS → PD: β = −0.138, *SE* = 0.039, *t* = 3.514, *p* < 0.001), supporting H7d. Collectively, these results suggest that stronger perceived support reduces the extent to which digitally coordinated learning experiences are associated with psychological distress.

In addition, PSup significantly moderated the relationship between PD and PWB (PS × PD → PWB: β = −0.184, *SE* = 0.031, *t* = 5.989, *p* < 0.001). In statistical terms, this confirms a significant moderation effect consistent with H8. That said, because the direct PD → PWB effect in your Table 6 is positive (β = 0.329), the negative interaction term implies that as PSup increases, the positive slope of PD → PWB becomes weaker (i.e., the PD–PWB association is attenuated at higher support). This is compatible with your “weakening” framing, but you may want to ensure the sign logic aligns with your theoretical interpretation of “detrimental effect” in H8.

**Figure 3:**
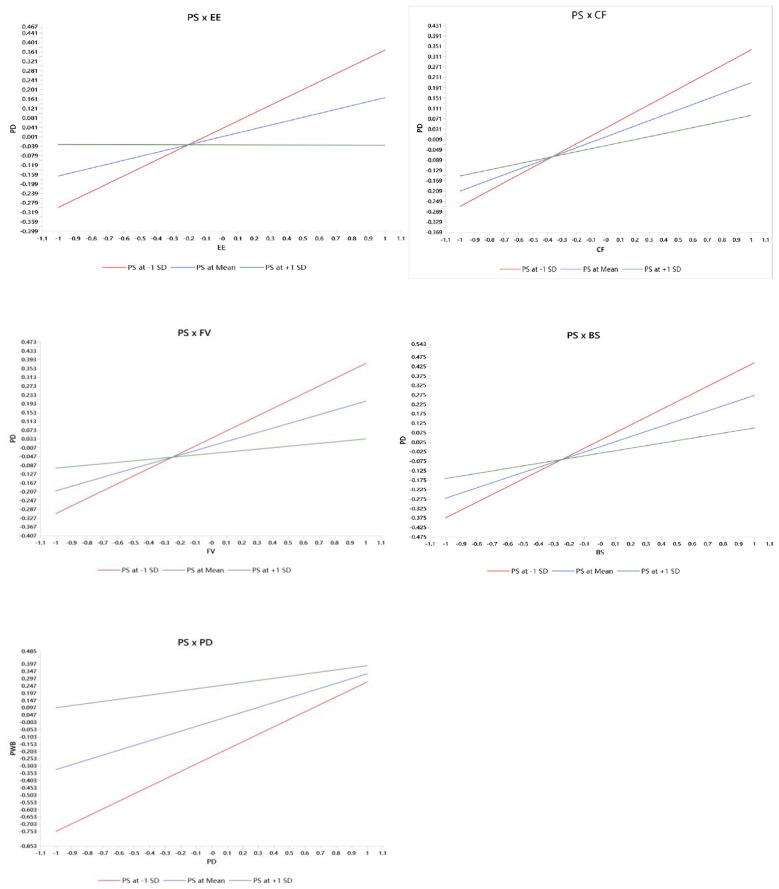
Simple slope plots.

To further interpret the significant moderation effects, we plotted simple slopes at low (−1 SD), mean, and high (+1 SD) levels of perceived support (PSup; Figure 3). As shown in the interaction plots (PSup × EE/CF/FV/BS → PD), the positive associations between each IMCLE cue (EE, CF, FV, and BS) and psychological distress (PD) are steepest when PSup is low and become progressively flatter as PSup increases. This consistent pattern indicates a buffering effect: higher perceived support attenuates the extent to which stronger coordinated learning experiences are accompanied by higher distress, aligning with the negative interaction coefficients reported in Table 7. In addition, the PSup × PD plot shows that the PD–psychological well-being (PWB) slope is strongest at low PSup and weaker at high PSup, suggesting that perceived support also dampens the marginal effect of PD on PWB, which is consistent with the negative PSup × PD interaction term in Table 7.

Finally, Table 7 reports significant moderated indirect effects through PD, indicating that PSup conditions the PD-mediated pathway for each IMCLE dimension. Specifically, the moderated indirect effects were significant for EE (PS × EE → PD → PWB: β = −0.055, *SE* = 0.013, *t* = 4.114, *p* < 0.001), CF (β = −0.031, *SE* = 0.012, *t* = 2.462, *p* = 0.014), FV (β = −0.045, *SE* = 0.014, *t* = 3.324, *p* = 0.001), and BS (β = −0.045, *SE* = 0.014, *t* = 3.308, *p* = 0.001). These negative coefficients suggest that higher perceived support weakens the indirect effect operating through psychological distress, consistent with the buffering logic proposed in H7a–H7d.

### ANN results

Building on the PLS-SEM analysis, this study further incorporated artificial neural networks (ANN) to enhance the predictive precision of the model regarding learning behaviors and psychological well-being. As an information-processing model that simulates the functioning of the human nervous system, ANN can effectively capture nonlinear and non-compensatory patterns in variable relationships, making it particularly suitable for identifying complex causal structures and potential interaction effects (Ferasso and Alnoor, 2022).

Specifically, this study employed a feedforward multilayer perceptron (MLP) architecture consisting of an input layer, a single hidden layer, and an output layer, and trained the network with the backpropagation (BP) algorithm to optimize parameters. A key advantage of ANN is that it does not rely on conventional normality or linearity assumptions; instead, it can automatically learn complex relational structures from the data. This capability allows ANN to effectively complement PLS-SEM, especially in identifying nonlinear factors that shape students' learning behaviors and psychological well-being. Using ANN, the model can estimate the relative importance of antecedent variables, thereby further strengthening the predictive power for psychological well-being and learning behaviors.

In this study, the ANN training results provide an importance ranking of the latent variables and reveal the relative weights of different predictors within the learning process, offering data-driven support for optimizing learning environments and designing targeted interventions.

**Figure 4:**
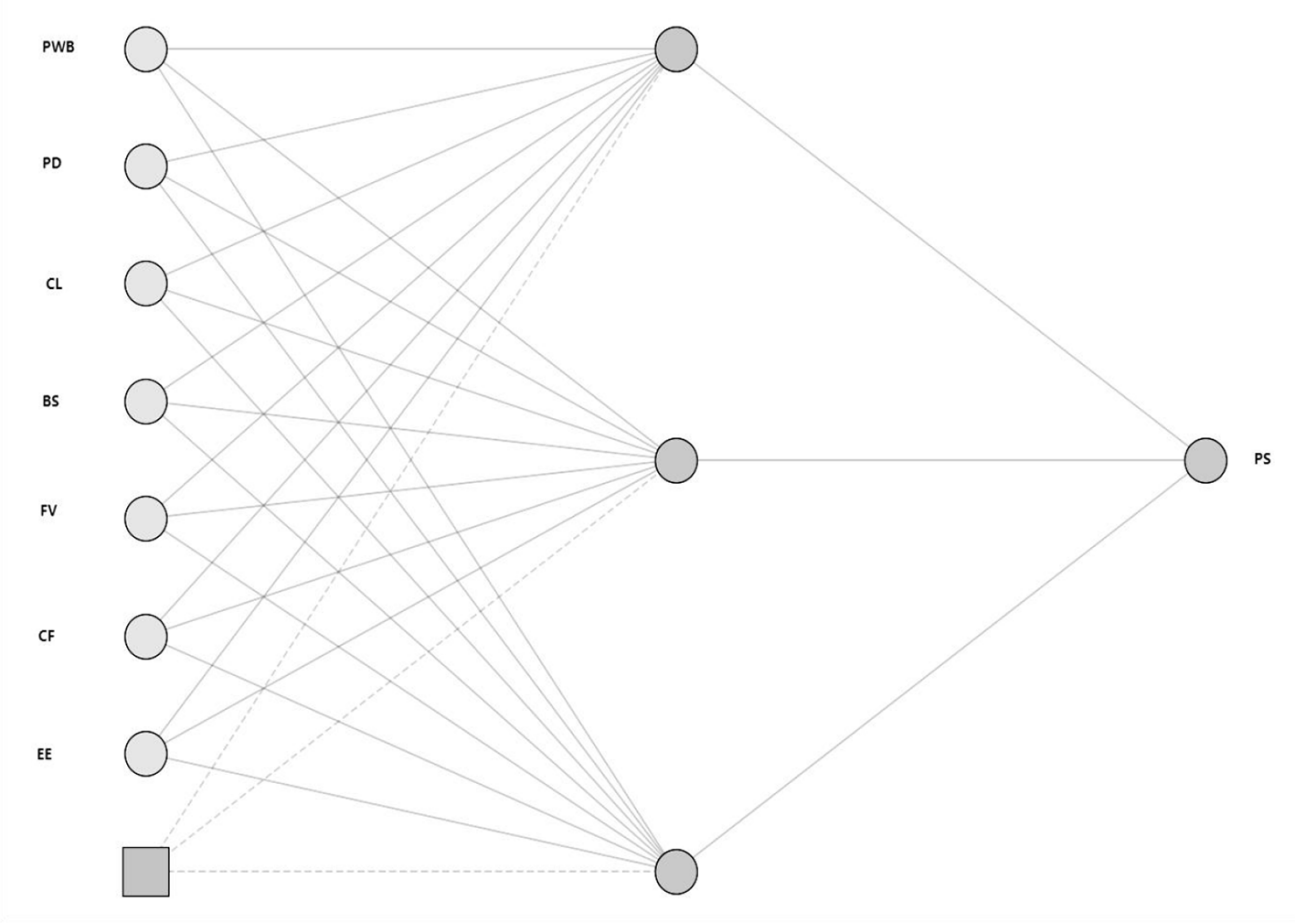
ANN model.

As shown in Figure 4, this study constructed a multilayer feedforward neural network (MLP) trained with the backpropagation (BP) algorithm. The input layer comprised multiple predictors of students' psychological well-being—namely empowerment effectiveness, collaboration facilitation, feedback visibility, and boundary safety—while the output layer was psychological well-being (PWB). The model adopted a three-layer architecture with one hidden layer, and a sigmoid activation function was applied to capture nonlinear relationships among variables. All input variables were standardized prior to training. To enhance generalizability and reduce the risk of overfitting, ten-fold cross-validation was used to split the data into training and testing sets. After model training, sensitivity analysis was conducted to evaluate the contribution of each input variable to the predicted outcome, providing an important benchmark for subsequent comparisons with the PLS-SEM path-effect results.

As reported in Table 8, across the 10 neural network models, the mean RMSE for the training sets was 0.102 and the mean RMSE for the testing sets was 0.100; the standard deviations were both 0.01, indicating very small fluctuations. These results suggest that the model exhibited highly consistent performance across folds, with no evidence of overfitting or underfitting. Both training and testing errors remained at low levels, demonstrating that the proposed ANN model has strong predictive capability and stable generalizability, and is therefore well suited for predictive analysis.

**Table 8:** Predictive accuracy of the ANN model.

**Neural network**	Training		Testing	
	***N***	**RMSE**	***N***	**RMSE**
ANN1	289	0.120	111.000	0.111
ANN2	287	0.119	113.000	0.102
ANN3	283	0.103	117.000	0.112
ANN4	285	0.112	115.000	0.100
ANN5	284	0.114	116.000	0.108
ANN6	282	0.120	118.000	0.104
ANN7	288	0.114	112.000	0.104
ANN8	281	0.111	119.000	0.107
ANN9	290	0.111	110.000	0.117
ANN10	286	0.110	114.000	0.115
Mean		0.113		0.108
SD	0.01		0.01	

According to Table 9, the ANN model generated a ranking of the relative importance of the antecedent variables. The results show that collaborative learning (CL) contributed most to the prediction, followed by collaboration facilitation (CF) and empowerment effectiveness (EE). Together, these three variables constitute the primary sources of predictive power for psychological well-being (PWB). Feedback visibility (FV) and psychological distress (PD) demonstrated a moderate level of contribution, whereas perceived support (PSup) and boundary safety (BS) contributed relatively less, with boundary safety (BS) emerging as the weakest predictor.

**Table 9:** Sensitivity analysis.

**Neural network**	**CF**	**CL**	**EE**	**FV**	**PD**	**PS**	**BS**
ANN1	0.3406	0.2956	0.3083	0.3621	0.3304	0.3792	0.2404
ANN2	0.3393	0.3806	0.2825	0.3327	0.2541	0.3582	0.2189
ANN3	0.3283	0.3024	0.353	0.2632	0.2834	0.3751	0.3402
ANN4	0.3715	0.3386	0.2738	0.2733	0.3541	0.3143	0.3475
ANN5	0.2918	0.3439	0.2636	0.3525	0.3298	0.3853	0.2355
ANN6	0.3376	0.3861	0.3991	0.2602	0.3671	0.26	0.2542
ANN7	0.3664	0.2997	0.356	0.3963	0.2773	0.3793	0.3145
ANN8	0.3559	0.3845	0.3298	0.2963	0.3709	0.2527	0.2907
ANN9	0.3933	0.3624	0.2717	0.2777	0.294	0.3701	0.3406
ANN10	0.3577	0.3501	0.3649	0.3849	0.3945	0.2869	0.219
Mean relative importance	0.34824	0.34439	0.32027	0.31992	0.32556	0.33611	0.28015
Normalized relative importance	73.20%	78.88%	70.35%	72.30%	76.22%	75.87%	72.90%

As shown in Table 10, the overall importance ranking is consistent with the PLS-SEM path coefficient results. This indicates that, in predicting psychological well-being, collaborative learning (CL), collaboration facilitation (CF), and psychological distress (PD) make the largest contributions, while boundary safety (BS) plays a comparatively smaller role in the model. Specifically, CL consistently ranks among the top predictors, highlighting its strongest predictive capability for enhancing psychological well-being. CF and PD also exhibit substantial influence. By contrast, BS ranks lower, suggesting a weaker role in predicting psychological well-being. This pattern aligns with the path coefficients obtained from the PLS-SEM analysis, further supporting the robustness and reliability of the proposed model and providing strong evidence for subsequent analyses and practical applications.

**Table 10:** Comparison between PLS-SEM and ANN results.

**Path**	**SEM path coefficient**	**ANN importance (%)**	**Path coefficient rank**	**Importance rank**
CL -> PWB	0.352436732	0.7888	1	1
PD -> PWB	0.328663708	0.7622	2	2
PS -> PWB	0.240211614	0.7587	3	3
CF -> PWB	0.185925336	0.732	4	4
BS -> PWB	0.165495114	0.729	5	5
FV -> PWB	0.160724637	0.723	6	6
EE -> PWB	0.15267861	0.7035	7	7

#### NCA results

Based on the results reported in Table 11, this study employed necessary condition analysis (NCA) to assess the necessity of each latent variable. The findings indicate that collaborative learning (CL), empowerment effectiveness (EE), feedback visibility (FV), and boundary safety (BS) all exhibited high necessity accuracy (close to 100%). Moreover, their ceiling zones, scope/range, and effect sizes suggested strong necessity impacts. In particular, CL and EE stood out with 100% accuracy, implying that they function as indispensable conditions for achieving higher levels of psychological well-being (PWB). Collaboration facilitation (CF) also demonstrated relatively high necessity accuracy, further underscoring its important role in collaborative learning and psychological well-being.

**Table 11:** NCA results.

**Condition**	**Method**	**Accuracy (%)**	**Ceiling zone**	**Range**	**Effect size (*d*)**	***p*-value**
CF	CE	100.00	0.001	0.970	0.001	0.129
	CR	96.00	0.001	0.995	0.000	0.045
CL	CE	100.00%	0.000	0.986	0.000	0.074
	CR	98.00	0.001	0.999	0.001	0.031
EE	CE	100.00	0.002	0.979	0.002	0.167
	CR	97.00	0.001	0.984	0.000	0.032
FV	CE	100.00	0.000	0.993	0.003	0.106
	CR	97.00	0.000	0.993	0.001	0.186
PD	CE	100.00	0.001	0.985	0.002	0.090
	CR	97.00	0.001	0.998	0.001	0.173
BS	CE	100.00	0.001	0.998	0.001	0.171
	CR	97.00	0.000	0.994	0.002	0.169
PS	CE	100.00	0.000	0.994	0.002	0.148
	CR	0.98	0.001	0.980	0.000	0.052

By contrast, psychological distress (PD) and perceived support (PSup) showed slightly lower necessity accuracy, yet they still exhibited a meaningful degree of influence.

In addition, boundary safety (BS) achieved an accuracy level close to 100%, suggesting that clear rules and a sense of psychological safety are critical in the formation of psychological well-being. The influence of psychological distress (PD) on psychological well-being was further corroborated; its accuracy and effect size also indicate that reducing psychological distress may contribute to improving students' psychological well-being.

Overall, these results suggest that NCA enables the identification of key necessary factors for psychological well-being and provides actionable evidence for subsequent managerial decisions. This can help universities optimize resource allocation in digital learning environments, enhance learning experiences, and ultimately promote students' psychological well-being.

As generative artificial intelligence (GAI) and social media platforms become increasingly embedded in higher education learning contexts, students' learning patterns and psychological experiences have undergone substantial changes. In digital learning environments in particular, the collaborative form of “individual–tool–platform–group” learning is gradually replacing the traditional linear model of “individual–textbook” learning. Students not only use GAI for tutoring, planning, and problem solving, but also rely on social media for learning-process management, peer interaction, and resource sharing. This shift generates more complex learning dynamics and psychological interplay, spanning learning efficiency, affective experience, and mental health. Against this backdrop, the Intelligent Media–Coordinated Learning Experience (IMCLE) framework proposed in this study seeks to explain how students' psychological well-being (PWB) is shaped in learning situations where GAI and social media are intertwined, through the interaction between perceived empowerment and perceived burden.

## Discussion

The results indicate that the four dimensions of IMCLE—empowerment effectiveness (EE), collaboration facilitation (CF), feedback visibility (FV), and boundary safety (BS)—exert significant effects on collaborative learning (CL) and psychological well-being (PWB). Notably, empowerment effectiveness and collaboration facilitation show the strongest positive effects on collaborative learning, which in turn promotes higher psychological well-being (Chaudhry et al., 2024; Liu et al., 2022). As a mediator, collaborative learning strengthens learning interaction and resource sharing, thereby enhancing students' engagement and sense of accomplishment. In contrast, the role of boundary safety appears comparatively weaker, which may reflect that students are not yet sufficiently attentive to privacy and data-security risks in digital learning settings, making boundary-related cues less salient in their well-being formation (Chen and Chen, 2025).

Regarding psychological distress (PD), the findings reveal a pattern that differs from the conventional “stress depletion → reduced well-being” narrative (Duc, 2026; Lin and Lachman, 2024). Specifically, the IMCLE dimensions significantly increased PD, and the direct association between PD and PWB was positive in the structural model, leading H4 to be unsupported. This suggests that in highly coordinated GAI–social media learning contexts, distress may function more as an activation signal accompanying intensive engagement (e.g., heightened demands, time pressure, and evaluative visibility) rather than purely as an erosive factor (Sparks et al., 2025). In other words, students may experience elevated strain while simultaneously perceiving greater progress, efficacy, or accomplishment, yielding a co-occurrence of “pressure and growth” in digitally coordinated learning (Podsakoff et al., 2023).

Mechanistically, the dual-path model therefore points to a more nuanced process in which resource gain via collaborative learning coexists with a strain component reflected in psychological distress. While CL consistently conveys positive gains to well-being, the PD pathway shows significant indirect effects whose direction is opposite to the “negative mediation” wording in H6a–H6d. This inconsistency indicates that the PD construct, as operationalized in this study, may capture a form of high-engagement stress that does not necessarily undermine well-being in this context. This finding invites future work to further differentiate between distress that is depleting vs. distress that is engagement-linked in digitally mediated learning environments.

Importantly, perceived support (PSup) operates as a boundary condition shaping these relationships. Consistent with the buffering logic, PSup significantly weakened the positive links between IMCLE dimensions and PD (H7a–H7d). Moreover, PSup also moderated the PD–PWB relationship: as perceived support increased, the positive association between PD and PWB became weaker, indicating an attenuation effect at higher support levels. Together, these results imply that supportive environments can reduce the extent to which coordinated learning experiences translate into heightened strain, and they also reshape how strain relates to well-being under different support conditions (Hou et al., 2025).

The ANN analysis further corroborates the predictive relevance of IMCLE dimensions for psychological well-being. Sensitivity analysis indicates that empowerment effectiveness (EE) and collaboration facilitation (CF) contribute most strongly to the prediction of psychological well-being, whereas boundary safety (BS) contributes less. By capturing nonlinear relationships among IMCLE dimensions, the ANN approach reveals complex patterns that may not be fully reflected in the PLS-SEM results, strengthening predictive precision.

Finally, necessary condition analysis (NCA) suggests that perceived support (PSup) plays an important role in shaping students' well-being formation. When perceived support is high, students appear better positioned to manage coordination-related demands, and the overall IMCLE–well-being linkage becomes more favorable.

### Theoretical contributions

**First**, this study introduces and validates Intelligent Media–Coordinated Learning Experience (IMCLE) as a theory-driven lens for explaining learning and mental-health outcomes in intertwined GAI–social media environments. By shifting the analytical focus from usage intensity to coordination quality, IMCLE captures how students experience the joint functioning of tool affordances, platform visibility, and interaction structures. The four experiential cues—empowerment effectiveness, collaboration facilitation, feedback visibility, and boundary safety—jointly predict collaborative learning and well-being, offering a more actionable conceptualization for digitally mediated learning than behavior-only indicators.

**Second**, the findings refine dominant “stress depletion” assumptions by revealing an engagement-linked strain pattern in highly coordinated learning contexts. While IMCLE consistently promotes collaborative learning and thereby enhances well-being, IMCLE also increases psychological distress, and the structural model shows a positive PD–PWB association, rendering the hypothesized negative link unsupported. This sign pattern suggests that, under conditions of intensified demands, time pressure, and evaluative visibility, distress may function as an activation signal accompanying high engagement and perceived progress rather than purely as an erosive state. Theoretical implication is that digitally coordinated learning may produce a co-occurrence of “pressure and growth,” and future models should distinguish between depleting distress and activation/effort-linked strain instead of treating distress as uniformly detrimental.

**Third**, by integrating parallel gain and strain channels, the study advances a dual-path account of digital learning outcomes. Collaborative learning operates as a robust gain mechanism linking coordination quality to well-being, whereas the distress pathway is statistically meaningful but directionally inconsistent with the original “negative mediation” wording. This mismatch is theoretically informative: it indicates that the meaning of “distress” may be context-sensitive in digitally visible, high-interaction learning settings, and it motivates future theorizing that differentiates strain types (e.g., overload-driven vs. goal-striving strain) and tests boundary conditions under which each pathway becomes beneficial or harmful.

**Fourth**, the study extends support-based theorizing by showing that perceived support (PSup) is not merely a background resource but a structural boundary condition that reshapes both (a) how coordination translates into distress, and (b) how distress relates to well-being. The moderation and simple-slope patterns demonstrate a consistent buffering effect: higher PSup flattens the escalation of distress associated with stronger coordinated experience. Moreover, PSup attenuates the PD–PWB slope, implying that support environments influence the functional meaning of strain—i.e., whether strain escalates with coordination and how strongly it “spills over” into well-being outcomes. This contributes a more interaction-specific account of support in digitally mediated learning.

**Fifth**, methodologically, the study strengthens theory-building by adopting an explanation–prediction–necessity evidence chain. PLS-SEM clarifies the nomological structure and boundary effects; ANN complements this by ranking predictor importance and accommodating nonlinear contributions, highlighting empowerment effectiveness and collaboration facilitation as the most influential levers; and NCA adds threshold-oriented insight by indicating that well-being formation is contingent on meeting key baseline conditions (notably support-related). Together, this integrated approach advances a more complete theoretical understanding of digital well-being by linking “what explains,” “what predicts most,” and “what must be present” in coordinated learning contexts.

Overall, by formalizing IMCLE and demonstrating how gain processes, engagement-linked strain, and support contingencies jointly shape psychological well-being, this study provides a more realistic and generalizable theoretical account of students' well-being in digitally intelligent learning environments and offers a foundation for future work to refine strain typologies and contextual boundary conditions in GAI-enabled learning.

### Practical contributions

The findings yield actionable recommendations for universities and education administrators seeking to govern GAI–social media–enabled learning while protecting student well-being.

**First, Set minimum standards for “coordination quality” (IMCLE) in GAI-enabled learning**. Universities can translate IMCLE into operational requirements when approving tools/platforms and designing courses. For empowerment effectiveness (EE), require task-level usefulness (e.g., course-specific prompt templates, verified examples, and “when-not-to-use” guidance) and provide short onboarding modules to improve controllability. For collaboration facilitation (CF), adopt structured collaboration protocols (role assignment, milestones, peer-review rubrics) and embed them into the platform workflow rather than leaving collaboration to *ad-hoc* group chats. For feedback visibility (FV), implement traceable feedback loops (clear criteria, version history, response deadlines) so that students can see what changed and why, reducing repeated trial-and-error.

**Second, Build “feedback governance” to reduce uncertainty and evaluative pressure**. Given the salience of feedback mechanisms, universities should standardize feedback rules across courses: publish assessment criteria early, limit excessive public ranking/comparison features, and require instructors to use consistent feedback formats (e.g., “strengths–issues–next steps”). Where social media is used for learning, establish moderation norms (acceptable posting, respectful critique) and a rapid-response channel for conflict or misinformation.

**Third, Manage distress as a governance target: monitor load and introduce early-warning support**. Because coordinated learning can intensify demands and time pressure, institutions should introduce lightweight monitoring and safeguards: periodic short check-ins (e.g., two-item strain screening), workload coordination across major deadlines, and “cool-down” policies (quiet periods, optional anonymity in peer feedback). For high-intensity weeks, provide structured coping resources (micro-workshops on time management, attention recovery, and social comparison management) rather than only offering general counseling.

**Fourth, Institutionalize perceived support (PSup) through multi-layer support infrastructure**. Since PSup buffers the link between coordinated experience and distress, universities should make support visible and quickly accessible. Actionable steps include: (a) a dedicated helpdesk for GAI/platform issues (technical + learning strategy), (b) peer mentor programs (trained student mentors for tool use and group coordination), and (c) instructor “office-hour guarantees” during key project phases. Critically, support should be routinized (clear entry points and response time expectations), not left to informal networks.

**Fifth, Prioritize interventions using predictive importance and minimum-condition logic**. To allocate limited resources, administrators can use the study's predictive evidence to prioritize: first improve EE and CF (highest leverage), then strengthen FV through structured feedback loops, while treating boundary safety as a necessary “baseline hygiene” factor via privacy guidance, consent rules, and data-protection prompts. This sequencing helps universities invest first in the most influential levers while ensuring minimum safeguards are in place.

**Sixth, Establish governance and accountability: define roles and measurable indicators**. Finally, universities should assign clear responsibility (e.g., teaching affairs office + IT + counseling center + program directors) and track indicators such as perceived support availability, feedback timeliness, group collaboration quality, and strain escalation during peak periods. Making these indicators part of routine quality assurance can improve both learning performance and student well-being while supporting the sustainable adoption of GAI-enabled learning.

### Limitations and future research

This study offers several unique contributions—most notably, the IMCLE framework that shifts attention from technology use to coordination-quality experience in intertwined GAI–social media learning, and an explanation–prediction–necessity evidence chain (PLS-SEM, ANN, NCA) that identifies both high-leverage cues and baseline conditions. Empirically, the findings show that IMCLE cues reliably promote collaborative learning, which in turn enhances psychological well-being, while perceived support consistently buffers the coordination–distress links. At the same time, the positive PD-related sign pattern suggests that strain may be engagement-linked in highly coordinated learning, indicating the need to refine how “distress” is theorized and measured in digitally visible learning contexts. These results carry implications for practice: universities should prioritize empowerment and collaboration affordances, strengthen feedback governance, and institutionalize support structures that prevent coordination-related strain from escalating.

Despite these contributions, several limitations warrant further investigation. First, the sample was drawn primarily from university students in a limited geographic scope; regional and institutional differences in digital literacy, platform norms, and learning demands may constrain generalizability. Future studies should replicate the model across regions, disciplines, institutions, and learning modes, and test cross-cultural robustness. Second, although we used a two-wave design to reduce common method bias, the data remain essentially observational and cannot fully capture dynamic within-person fluctuations. Longitudinal and experience-sampling designs, ideally combined with behavioral traces (e.g., platform logs, feedback timestamps, collaboration records), would strengthen causal inference and clarify how coordination quality and strain co-evolve over time. Third, our results suggest that the “distress” pathway may not uniformly represent depletion; future research should differentiate depleting distress from activation/effort-linked strain by incorporating stressor typologies (e.g., challenge vs. hindrance), workload/visibility conditions, and task types (routine assignments vs. high-stakes projects). Fourth, future work could expand boundary conditions by integrating individual-difference factors such as technology anxiety, privacy concerns, trust, and self-regulation, and by comparing different platform designs and pedagogical arrangements to identify when boundary safety becomes more salient. Finally, while ANN and NCA complement PLS-SEM, these analyses rely on modeling choices and a single dataset. Replications with larger samples, alternative machine-learning approaches, and multi-source data are needed to verify robustness and improve the portability of intervention priorities and threshold-based recommendations.

## Data Availability

The original contributions presented in the study are included in the article/Supplementary material, further inquiries can be directed to the corresponding author/s.
